# Ventilation Improvement Strategies Among K–12 Public Schools — The National School COVID-19 Prevention Study, United States, February 14–March 27, 2022

**DOI:** 10.15585/mmwr.mm7123e2

**Published:** 2022-06-10

**Authors:** Sanjana Pampati, Catherine N. Rasberry, Luke McConnell, Zach Timpe, Sarah Lee, Patricia Spencer, Shamia Moore, Kenneth R. Mead, Colleen Crittenden Murray, Xiaoyi Deng, Ronaldo Iachan, Tasneem Tripathi, Stephen B. Martin, Lisa C. Barrios

**Affiliations:** ^1^Division of Adolescent and School Health, National Center for HIV, Viral Hepatitis, STD, and TB Prevention, CDC; ^2^CDC COVID-19 Emergency Response Team; ^3^ICF, Atlanta, Georgia; ^4^Division of Population Health, National Center for Chronic Disease Prevention and Health Promotion, CDC; ^5^Oak Ridge Institute for Science and Education, Oak Ridge, Tennessee; ^6^Division of Field Studies and Engineering, National Institute for Occupational Safety and Health, CDC; ^7^Respiratory Health Division, National Institute for Occupational Safety and Health, CDC

Effective COVID-19 prevention in kindergarten through grade 12 (K–12) schools requires multicomponent prevention strategies in school buildings and school-based transportation, including improving ventilation (*1*). Improved ventilation can reduce the concentration of infectious aerosols and duration of potential exposures (*2*,*3*), is linked to lower COVID-19 incidence (*4*), and can offer other health-related benefits (e.g., better measures of respiratory health, such as reduced allergy symptoms) (*5*). Whereas ambient wind currents effectively dissipate SARS-CoV-2 (the virus that causes COVID-19) outdoors,* ventilation systems provide protective airflow and filtration indoors (*6*). CDC examined reported ventilation improvement strategies among a nationally representative sample of K–12 public schools in the United States using wave 4 (February 14–March 27, 2022) data from the National School COVID-19 Prevention Study (NSCPS) (420 schools), a web-based survey administered to school-level administrators beginning in summer 2021.^†^ The most frequently reported ventilation improvement strategies were lower-cost strategies, including relocating activities outdoors (73.6%), inspecting and validating existing heating, ventilation and air conditioning (HVAC) systems (70.5%), and opening doors (67.3%) or windows (67.2%) when safe to do so. A smaller proportion of schools reported more resource-intensive strategies such as replacing or upgrading HVAC systems (38.5%) or using high-efficiency particulate air (HEPA) filtration systems in classrooms (28.2%) or eating areas (29.8%). Rural and mid-poverty–level schools were less likely to report several resource-intensive strategies. For example, rural schools were less likely to use portable HEPA filtration systems in classrooms (15.6%) than were city (37.7%) and suburban schools (32.9%), and mid-poverty–level schools were less likely than were high-poverty–level schools to have replaced or upgraded HVAC systems (32.4% versus 48.8%). Substantial federal resources to improve ventilation in schools are available.^§^ Ensuring their use might reduce SARS-CoV-2 transmission in schools. Focusing support on schools least likely to have resource-intensive ventilation strategies might facilitate equitable implementation of ventilation improvements.

NSCPS is an ongoing population-based, longitudinal study that explores implementation and effectiveness of COVID-19 prevention strategies in a representative sample of U.S. K–12 public schools. The sampling frame consists of all public schools in the 50 states and the District of Columbia and includes a combination of Common Core Data from the National Center for Education Statistics (NCES) and Market Data Retrieval database.^¶^^,^** A web-based survey is administered to school administrators (e.g., principals) or school-level designees familiar with COVID-19 prevention strategies (e.g., nurses) at the eligible school. Recruitment involves emails and telephone calls to potential participants at eligible schools. A stratified random sample of schools by region, school level, and NCES locale is conducted.^††^ The final sample includes 1,602 schools.^§§^

Data from survey wave 4 (420 schools; response rate = 26%), collected during February 14–March 27, 2022, were weighted to account for nonresponse and design strata. This study examined 11 ventilation improvement strategies in schools and on school-based transportation. The percentage of students eligible for free or reduced-priced meals during 2019–20 served as a proxy for school poverty level (*7*). Low-, mid-, and high-poverty schools were defined as schools with ≤25%, 26%–75%, and ≥76% of students, respectively, eligible for free or reduced-priced meals.^¶¶^ School locale was categorized as city, suburban, town, or rural according to NCES.***

Weighted percentages (with 95% CIs) of ventilation improvement strategies among K–12 public schools, including by locale and school poverty level, were estimated. Chi-square tests were used to test for differences in the percentage of schools reporting each ventilation strategy by school-level characteristics; p-values <0.05 were considered statistically significant. Analyses were conducted using R (version 4.1.2; R Foundation). This activity was reviewed by CDC and conducted consistent with applicable federal law and CDC policy.^†††^ The study was reviewed and approved by ICF’s Institutional Review Board.^§§§^

Among 11 ventilation improvement strategies assessed, the four most frequently reported were relocating activities outdoors when possible (73.6%), having existing HVAC systems inspected and validated since the start of the pandemic (70.5%), and opening doors (67.3%) and windows (67.2%) when safe to do so (Table 1). The least frequently reported strategies were using portable HEPA filtration systems in classrooms (28.2%), using HEPA filtration systems in areas where students eat (29.8%), using portable HEPA filtration systems for high-risk areas (32.8%), using fans to increase effectiveness of windows opened when safe to do so (37.0%), and having replaced or upgraded HVAC systems since the beginning of the pandemic (38.5%).

**TABLE 1:** Strategies to improve ventilation in U.S. kindergarten through grade 12 public schools (N = 420) — National School COVID-19 Prevention Study, wave 4, United States, February 14–March 27, 2022

Strategies (no.)^†^	% (95% CI)*		
	Yes	No	Don’t know
**Implemented since the start of the COVID-19 pandemic** ^§^			
Inspected and validated existing HVAC systems for cleanliness, function, and code-compliant operation (403)	70.5 (65.6–75.1)	7.1 (4.8–10.5)	22.3 (18.3–26.9)
Replaced/Upgraded HVAC systems (403)	38.5 (33.6–43.6)	33.9 (29.1–39.0)	27.7 (23.4–32.4)
**Implemented currently** ^¶^			
Open doors to hallway or outside when safe to do so (403)	67.3 (62.3–71.9)	29.4 (24.9–34.4)	3.3 (1.9–5.8)
Open windows when safe to do so (404)	67.2 (62.2–71.8)	29.5 (24.9–34.6)	3.3 (1.9–5.7)
Use fans to increase effectiveness of open windows when safe to do so (404)	37.0 (32.1–42.1)	57.3 (52.1–62.4)	5.7 (3.7–8.6)
Relocate activities to outdoors when possible (404)	73.6 (68.7–78.0)	23.3 (19.0–28.3)	—**
Increase ventilation in areas where students eat (403)	43.0 (37.9–48.3)	46.6 (41.2–52.1)	10.4 (7.8–13.7)
Use HEPA filtration systems in areas where students eat (402)	29.8 (25.2–34.8)	48.6 (43.3–54.0)	21.6 (17.7–26.0)
Use portable HEPA filtration systems in classrooms (404)	28.2 (24.0–32.8)	58.2 (53.1–63.1)	13.6 (10.5–17.6)
Use portable HEPA filtration systems for high-risk areas^††^ (403)	32.8 (28.0–38.0)	54.0 (48.7–59.2)	13.2 (10.0–17.2)
Open windows on school buses (361)	63.6 (58.1–68.7)	8.9 (6.4–12.3)	27.5 (22.9–32.8)

**Abbreviations:** HEPA = high-efficiency particulate air; HVAC = heating, ventilation, and air conditioning.

* Weighted percentages and 95% CIs are presented for each category. The following responses were categorized as missing and excluded from analyses: “Not applicable, my school has been virtual since the start of the pandemic” for survey questions assessing ventilation strategies implemented since the start of the COVID-19 pandemic; “Not applicable, my school is currently virtual” for survey questions assessing ventilation strategies implemented currently; and “Not applicable, our school does not use school buses” for strategies to improve ventilation in school-based transportation.

^†^ Unweighted count of schools with available data for each ventilation strategy.

^§^ Respondents were asked whether their school had implemented this measure “since the start of the COVID-19 pandemic.”

^¶^ Respondents were asked whether their school currently has this measure in place.

** Estimate was suppressed because the relative SE was >30%.

^††^ Examples include nurse’s office, isolation areas, or rooms where mask guidance is less likely to be followed.

Six ventilation strategies significantly differed by locale (Table 2).^¶¶¶^ City schools were less likely to report opening windows when safe to do so (53.9%) than were suburban (69.5%), town (75.3%), and rural (73.5%) schools; city schools also were less likely to use fans to increase effectiveness of opening windows when safe to do so (26.1%) than were town (43.0%) and rural (43.3%) schools. City schools were less likely to open windows on school buses (54.5%) than were rural schools (72.9%). Rural schools were less likely to use HEPA filtration systems in areas where students eat (19.1%) or to use portable HEPA filtration systems in classrooms (15.6%) than were city (33.4% and 37.7%, respectively) and suburban schools (33.2% and 32.9%, respectively). Rural schools were less likely than were city schools to use portable HEPA filtration systems for high-risk areas (22.0% versus 44.7%).

**TABLE 2:** Strategies to improve ventilation in kindergarten through grade 12 public schools by locale (N = 420) — National School COVID-19 Prevention Study, wave 4, United States, February 14–March 27, 2022

Strategies (no.)^¶^	NCES school locale,*^,†^ % (95% CI)^§^			
	City	Suburb	Town	Rural
Strategies implemented since the start of the COVID-19 pandemic**				
Inspected/Validated existing HVAC systems for cleanliness, function, and code-compliant operation (364)	72.1 (61.6–80.6)	76.4 (66.4–84.2)	67.5 (54.3–78.4)	65.6 (54.8–74.9)
Replaced/Upgraded HVAC systems (364)	42.8 (32.1–54.2)	42.8 (34.4–51.5)	39.2 (25.7–54.6)	29.7 (20.6–40.9)
**Strategies implemented currently** ^††^				
Open doors to hallway or outside when safe to do so (364)	63.4 (51.7–73.7)	63.8 (54.7–72.1)	63.8 (49.8–75.8)	73.2 (62.7–81.5)
Open windows when safe to do so (365)^§§,¶¶,^***	53.9 (42.8–64.6)	69.5 (60.3–77.5)	75.3 (60.2–86.0)	73.5 (63.1–81.8)
Use fans to increase effectiveness of open windows when safe to do so (365)^§§,^***	26.1 (17.4–37.2)	35.3 (26.2–45.5)	43.0 (30.2–56.9)	43.3 (34.0–53.1)
Relocate activities to outdoors when possible (365)	70.7 (59.2–80.1)	77.9 (68.1–85.3)	70.5 (58.9–79.9)	71.0 (60.7–79.6)
Increase ventilation in areas where students eat (364)	45.4 (34.4–56.9)	48.3 (38.8–57.9)	44.2 (31.7–57.4)	36.2 (26.1–47.7)
Use HEPA filtration systems in areas where students eat (363)^§§,†††^	33.4 (23.1–45.5)	33.2 (25.0–42.5)	27.4 (15.7–43.3)	19.1 (12.4–28.3)
Use portable HEPA filtration systems in classrooms (365)^§§,†††^	37.7 (28.2–48.4)	32.9 (25.1–41.7)	22.8 (13.4–36.1)	15.6 (9.7–24.0)
Use portable HEPA filtration systems for high-risk areas^§§§^ (364)^§§^	44.7 (33.2–56.8)	34.1 (25.6–43.8)	35.3 (22.8–50.2)	22.0 (14.3–32.3)
Open windows on school buses (325)^§§^	54.5 (41.4–66.9)	60.1 (49.9–69.5)	64.4 (50.1–76.5)	72.9 (62.8–81.0)

**Abbreviations:** HEPA = high-efficiency particulate air; HVAC = heating, ventilation, and air conditioning; NCES = National Center for Education Statistics.

* School locale was categorized based on the NCES locale classification scheme into four categories: city, suburb, town, or rural. https://nces.ed.gov/programs/edge/Geographic/LocaleBoundaries

^†^ No significant differences between rural versus town and suburb versus town schools were noted based on chi-square test (p>0.05).

^§^ Weighted percentages and 95% CIs of respondents indicating “yes” for each ventilation measure is reported. Respondents who indicated “no” or “don’t know” for each ventilation measure are combined and included in the denominator. The following responses were categorized as missing and excluded from analyses: “Not applicable, my school has been virtual since the start of the pandemic” for survey questions assessing ventilation strategies implemented since the start of the COVID-19 pandemic; “Not applicable, my school is currently virtual” for survey questions assessing ventilation strategies implemented currently; and “Not applicable, our school does not use school buses” for strategies to improve ventilation in school-based transportation.

^¶^ Unweighted count of schools with available data for each ventilation strategy and NCES school locale.

** Respondents were asked whether their school had implemented this measure “since the start of the COVID-19 pandemic.”

^††^ Respondents were asked whether their school currently had this measure in place.

^§§^ City schools differed significantly from rural schools based on chi-square test (p<0.05).

^¶¶^ Suburb schools differed significantly from city schools based on chi-square test (p<0.05).

*** City schools differed significantly from town schools based on chi-square test (p<0.05).

^†††^ Suburb schools differed significantly from rural schools based on chi-square test (p<0.05).

^§§§^ Examples include nurse’s office, isolation areas, or rooms where mask guidance is less likely to be followed.

Six ventilation strategies significantly differed by school poverty level (Table 3). Mid-poverty schools were less likely than were low-poverty schools to have inspected and validated existing HVAC systems (66.0% versus 83.0%). Mid-poverty schools were less likely than were high-poverty schools to have replaced or upgraded HVAC systems (32.4% versus 48.8%), relocated activities outdoors when possible (69.1% versus 83.0%), and increased ventilation in areas where students eat (37.8% versus 55.4%). Mid-poverty schools were less likely to use portable HEPA filtration systems in classrooms (20.5%) and use portable HEPA filtration systems for high-risk areas (24.1%) than were low-poverty (43.8% and 49.8%, respectively) and high-poverty schools (36.0% and 44.7%, respectively).

**TABLE 3:** Strategies to improve ventilation in U.S. kindergarten through grade 12 public schools by school poverty level (N = 420) — National School COVID-19 Prevention Study, wave 4, United States, February 14–March 27, 2022

Strategies (no.)^¶^	School poverty level,*^,†^ % (95% CI)^§^		
	Low	Mid	High
Strategies implemented since the start of the COVID-19 pandemic**			
Inspected and validated existing HVAC systems for cleanliness, function, and code-compliant operation (375)^††^	83.0 (70.9–90.7)	66.0 (58.6–72.7)	74.8 (64.4–83.0)
Replaced/Upgraded HVAC systems (375)^§§^	45.3 (33.1–58.0)	32.4 (26.3–39.2)	48.8 (37.0–60.6)
**Strategies implemented currently** ^¶¶^			
Open doors to hallway or outside when safe to do so (375)	66.1 (52.9–77.2)	66.0 (59.0–72.4)	69.1 (57.1–78.9)
Open windows when safe to do so (376)	74.4 (61.6–84.0)	65.3 (58.0–72.0)	69.7 (57.6–79.5)
Use fans to increase the effectiveness of open windows when safe to do so (376)	32.8 (21.8–46.0)	37.2 (30.5–44.4)	34.6 (24.9–45.8)
Relocate activities to outdoors when possible (376)^§§^	74.6 (62.1–84.0)	69.1 (61.7–75.6)	83.0 (71.8–90.4)
Increase ventilation in areas where students eat (376)^§§^	42.1 (30.3–54.9)	37.8 (31.1–45.0)	55.4 (43.0–67.1)
Use HEPA filtration systems in areas where students eat (375)	36.5 (25.3–49.3)	24.8 (19.2–31.4)	35.1 (24.8–47.1)
Use portable HEPA filtration systems in classrooms (376)^††,§§^	43.8 (31.5–56.9)	20.5 (15.7–26.4)	36.0 (25.9–47.4)
Use portable HEPA filtration systems for high-risk areas*** (376)^††,§§^	49.8 (37.0–62.6)	24.1 (18.4–30.8)	44.7 (33.1–56.9)
Open windows on school buses (338)	74.3 (59.7–84.9)	59.7 (52.3–66.6)	64.3 (52.4–74.7)

**Abbreviations:** FRPM = free or reduced-price meals; HEPA = high-efficiency particulate air; HVAC = heating, ventilation, and air conditioning; NCES = National Center for Education Statistics.

* The percentage of students eligible for FRPM during 2019–20 was used as a proxy for school poverty level. High-poverty schools were defined as public schools in which >75% of the students were eligible for FRPM, mid-poverty schools had 26%–75% students eligible for FRPM, and low-poverty schools had ≤25% students eligible for FRPM.

^†^ No significant differences between low- versus high-poverty schools were noted based on chi-square test (p>0.05).

^§^ Weighted percentages and 95% CIs of respondents indicating “yes” for each ventilation measure is reported. Respondents who indicated “no” or “don’t know” for each ventilation measure are combined and included in the denominator. The following responses were categorized as missing and excluded from analyses: “Not applicable, my school has been virtual since the start of the pandemic” for survey questions assessing ventilation strategies implemented since the start of the COVID-19 pandemic; “Not applicable, my school is currently virtual” for survey questions assessing ventilation strategies implemented currently; and “Not applicable, our school does not use school buses” for strategies to improve ventilation in school-based transportation.

^¶^ Unweighted count of schools with available data for each ventilation strategy and school poverty level.

** Respondents were asked whether their school had implemented this measure “since the start of the COVID-19 pandemic.”

^††^ Mid-poverty schools differed significantly from low-poverty schools based on chi-square test (p<0.05).

^§§^ Mid-poverty schools differed significantly from high-poverty schools based on chi-square test (p<0.05).

^¶¶^ Respondents were asked whether their school currently had this measure in place.

*** Examples include nurse’s office, isolation areas, or rooms where mask guidance is less likely to be followed.

## Discussion

These findings show differences in schools’ reported ventilation improvement strategies by school characteristics, including NCES locale and school poverty-level status. The study also found strategies that could be easily and affordably implemented (e.g., opening doors or windows when safe to do so) were among those most frequently reported and did not vary significantly by school poverty level. City schools were least likely to report strategies related to opening windows when safe to do so and might experience unique challenges that prohibit opening windows, including air and noise pollution, and limitations of the building (e.g., windows that cannot be opened). CDC’s COVID-19 guidance for schools (*1*) and for improving ventilation in buildings,**** as well as ASHRAE^††††^ guidance for schools and universities (*8*), emphasize numerous ways to improve ventilation, with strategies varying substantially in both financial cost and ease of implementation.^§§§§^

With regard to HVAC and HEPA filtration systems, having inspected and validated existing HVAC systems was reported as the only strategy used by a majority of schools. The other strategies related to HVAC and HEPA filtration systems require additional resources with varying costs. Differences by locale and school poverty level in implementing more resource-intensive strategies might be due to supply chain challenges, differences in school or community resources, or accessibility of technical assistance and support for applying to available sources of funding. NSCPS did not provide data on the funds schools used to implement these resource-intensive strategies; however, mid-poverty schools might have been least likely to implement these strategies because higher poverty schools might have had more experience in accessing and using federal funds, and lower poverty schools might have been able to implement some of these strategies without additional government support. Despite availability of substantial federal resources to improve ventilation in schools, findings suggest that additional efforts might be needed to ensure that all schools successfully access and use resources for ventilation improvements, particularly schools least likely to report using resource-intensive ventilation strategies (i.e., rural and mid-poverty schools). Public health professionals and funding agencies can support state and local education agencies and school districts by raising awareness about funding sources and ensuring their equitable distribution. Supplemental training and technical assistance can help schools identify and access applicable funding and understand what types of strategies can improve ventilation.

Strategies to improve ventilation are integral to CDC’s guidance for COVID-19 prevention in schools (*1*). Schools can work with local public health officials and monitor CDC COVID-19 community levels^¶¶¶¶^ to determine which prevention strategies might be needed based on their local context. Schools can put in place a core set of infectious disease prevention strategies, including optimizing ventilation and improving indoor air quality as part of normal operations. The addition of COVID-19–specific prevention strategies, including those that increase outdoor air intake and improve air filtration, can be tied to CDC COVID-19 community levels (*1*). In addition to preventing spread of COVID-19 and other infections, such as influenza (*9*), ventilation improvements implemented now might lead to broader and lasting improvements in the health of students and staff members. For example, improved ventilation has been linked to better measures of respiratory health (e.g., allergy symptoms), higher student performance, and decreased student absenteeism (*5*).

The findings in this report are subject to at least five limitations. First, presence of ventilation improvement strategies was assessed through a self-report survey, and responses might be influenced by social desirability bias or respondents’ level of awareness of ventilation-related strategies at the school. Second, ascertaining the knowledge and training of persons who completed the survey was not possible, and this might vary by school characteristics. Third, the survey response rate was low (26%); however, nonresponse weight adjustments were incorporated into analyses. Fourth, this study only identified respondents’ reports of strategies implemented to improve ventilation; it did not include direct measurements of the impact of those strategies (e.g., increased air flow). Finally, appropriate ventilation improvements likely vary by seasonality, environment, building type, and safety-related concerns; this study was not able to account for these distinctions.

Ventilation is a key strategy recommended to reduce COVID-19 spread in school settings. Ensuring use of ventilation improvement resources might reduce SARS-CoV-2 transmission in schools and also prevent transmission of other infectious diseases and lead to broader improvements in the health of students and staff members. Public health professionals can focus support on schools least likely to report using resource-intensive ventilation strategies to ensure more equitable implementation of ventilation strategies to reduce SARS-CoV-2 transmission.

**Summary**

**What is already known about this topic?**

School-based strategies to improve ventilation are associated with reduced incidence of COVID-19 in schools. Substantial federal resources are available to improve ventilation in schools.

**What is added by this report?**

Among a nationally representative sample of U.S. K–12 public schools, higher-cost and resource-intensive ventilation improvement strategies, such as using portable high-efficiency particular air (HEPA) filtration systems in classrooms were less frequently reported. Overall, rural and mid-poverty schools were the least likely to report implementing several resource-intensive ventilation strategies.

**What are the implications for public health practice?**

Ensuring use of ventilation improvement resources might reduce transmission of SARS-CoV-2 and other infectious diseases in schools. Focusing support on schools least likely to have implemented resource-intensive ventilation strategies might facilitate equitable implementation.

## Footnotes

*
https://www.medrxiv.org/content/10.1101/2020.10.03.20206110v6

†
https://www.cdc.gov/healthyyouth/data/nscps/index.htm

§ Federal funding for ventilation improvements in schools includes the U.S. Department of Education’s Elementary and Secondary School Emergency Relief Fund (https://oese.ed.gov/offices/education-stabilization-fund/elementary-secondary-school-emergency-relief-fund/), the Governor’s Emergency Education Relief Fund (https://oese.ed.gov/offices/education-stabilization-fund/governors-emergency-education-relief-fund/), the U.S. Department of Health and Humans Services’ FY 2021 American Rescue Plan Funding Increase for Head Start Programs funds (https://eclkc.ohs.acf.hhs.gov/policy/pi/acf-pi-hs-21-03), and the Epidemiology and Laboratory Capacity for Prevention and Control of Emerging Infectious Diseases Reopening Schools supplement (https://www.cdc.gov/ncezid/dpei/elc/covid-response/index.html).

¶ The following types of schools were excluded from the sampling frame: private, alternative, those run by the U.S. Department of Defense, and those with fewer than 30 students. In addition, schools providing services to a “pull-out” population in another eligible school were excluded from the sampling frame; for example, if students from an eligible school received specific vocational or educational services at a different school for a portion of the day, the latter school would not be included in the sampling frame.

** The Market Data Retrieval database provides information about individual U.S. schools, collating data from various other sources. https://mdreducation.com

†† A stratified random sample of schools was conducted using strata defined by U.S. Census region (Northeast, Midwest, South, and West), school level (elementary, middle, and high), and NCES locale (city, town, suburb, and rural). School level was categorized as elementary (included any grade from kindergarten through grade 4), middle (included either grade 7 or 8), or high (included any grade from 10 through 12). Schools assigned to more than one core level (e.g., K–8) were considered separate schools for sampling purposes.

§§ The first wave of data collection was administered during June–September 2021 to a subset of 600 schools and focused on the 2020–21 school year. The subsequent four waves of data collection, including wave 4 analyzed in this report, focus on the 2021–22 school year with a sample of 1,602 schools.

¶¶
https://nces.ed.gov/programs/coe/indicator/clb

***
https://nces.ed.gov/programs/edge/Geographic/LocaleBoundaries

††† 5 C.F.R. part 46.102(l)(2), 21 C.F.R. part 56; 42 U.S.C. Sect. 241(d); 5 U.S.C. Sect. 552a; 44 U.S.C. Sect. 3501 et seq.

§§§
https://www.icf.com

¶¶¶ Chi-square tests were used to identify differences in each ventilation strategy by NCES locale and school poverty level. Even when p-values were <0.05, 95% CIs for percentages by subgroup might overlap because of varying underlying statistical assumptions of chi-square tests compared with examining overlap of 95% CIs when determining statistical significance.

****
https://www.cdc.gov/coronavirus/2019-ncov/community/ventilation.html

†††† Formerly known as the American Society of Heating, Refrigerating and Air-Conditioning Engineers, the organization is now known as ASHRAE.

§§§§ Ventilation strategies examined have a range of initial and operating costs. Opening windows and doors when safe to do so and relocating activities outside likely have no associated costs. The cost of using fans to increase the effectiveness of open windows when safe to do so is estimated to be <$100 per unit. The cost of adding portable HEPA filtration systems is approximately $500 per unit. Additional information about estimated costs of ventilation strategies is available on CDC’s website for ventilation in buildings. https://www.cdc.gov/coronavirus/2019-ncov/community/ventilation.html

¶¶¶¶
https://www.cdc.gov/coronavirus/2019-ncov/science/community-levels.html
